# Fire-modulated fluctuations in nutrient availability stimulate biome-scale floristic turnover in time, and elevated species richness, in low-nutrient fynbos heathland

**DOI:** 10.1093/aob/mcad199

**Published:** 2023-12-27

**Authors:** G Anthony Verboom, Jasper A Slingsby, Michael D Cramer

**Affiliations:** Bolus Herbarium, University of Cape Town, Private Bag X3, Rondebosch, 7701, South Africa; Department of Biological Sciences, University of Cape Town, Private Bag X3, Rondebosch, 7701, South Africa; Department of Biological Sciences, University of Cape Town, Private Bag X3, Rondebosch, 7701, South Africa; Centre for Statistics in Ecology, Environment and Conservation, University of Cape Town, Private Bag X3, Rondebosch, 7701, South Africa; Fynbos Node, South African Environmental Observation Network (SAEON), Cape Town, South Africa; Department of Biological Sciences, University of Cape Town, Private Bag X3, Rondebosch, 7701, South Africa

**Keywords:** Alpha diversity, community richness, fire, temporal turnover, fynbos, resource availability, soil nutrients, species richness, postfire succession, vascular plants

## Abstract

**Background and Aims:**

In many systems, postfire vegetation recovery is characterized by temporal changes in plant species composition and richness. We attribute this to changes in resource availability with time since fire, with the magnitude of species turnover determined by the degree of resource limitation. Here, we test the hypothesis that postfire species turnover in South African fynbos heathland is powered by fire-modulated changes in nutrient availability, with the magnitude of turnover in nutrient-constrained fynbos being greater than in fertile renosterveld shrubland. We also test the hypothesis that floristic overlaps between fynbos and renosterveld are attributable to nutritional augmentation of fynbos soils immediately after fire.

**Methods:**

We use vegetation survey data from two sites on the Cape Peninsula to compare changes in species richness and composition with time since fire.

**Key Results:**

Fynbos communities display a clear decline in species richness with time since fire, whereas no such decline is apparent in renosterveld. In fynbos, declining species richness is associated with declines in the richness of plant families having high foliar concentrations of nitrogen, phosphorus and potassium and possessing attributes that are nutritionally costly. In contrast, families that dominate late-succession fynbos possess adaptations for the acquisition and retention of sparse nutrients. At the family level, recently burnt fynbos is compositionally more similar to renosterveld than is mature fynbos.

**Conclusions:**

Our data suggest that nutritionally driven species turnover contributes significantly to fynbos community richness. We propose that the extremely low baseline fertility of fynbos soils serves to lengthen the nutritional resource axis along which species can differentiate and coexist, thereby providing the opportunity for low-nutrient extremophiles to coexist spatially with species adapted to more fertile soil. This mechanism has the potential to operate in any resource-constrained system in which episodic disturbance affects resource availability.

## INTRODUCTION

Fire influences landscape-scale species richness in many ecosystems through its effect on vegetation structure and composition. In the moist tropics, for example, the presence of fire can switch the vegetation from a fire-averse forest state to a fire-prone grassland or savanna state, with the exclusion of fire having the reverse effect (Murphy and Bowman, 2012; Pausas and Keeley, 2014*a*; Pausas and Bond, 2020). Given that the spread of fire is influenced by multiple factors that vary across the landscape, fires tend to be patchy, and many tropical landscapes constitute grassland/savanna–forest mosaics (Murphy and Bowman, 2012; Das *et al.*, 2015; Staver *et al.*, 2017; Beckett and Bond, 2019). Importantly, given that grassland/savanna and forest communities comprise different species (Bond and Parr, 2010; Aleman *et al.*, 2020), this has implications for landscape-scale and regional species richness (Kelly and Brotons, 2017; Pausas and Ribeiro, 2017; Erdős *et al.*, 2018). In a similar manner, fire promotes the emergence of forest–shrubland (fynbos) mosaics in the Cape Floristic Region of South Africa (Coetsee *et al.*, 2015; Cramer *et al.*, 2019*a*; Slingsby *et al.*, 2020), whose cumulative species richness is enhanced by high floristic turnover between these vegetation types (Power *et al.*, 2017).

Besides facilitating landscape-scale coexistence of fire-averse and fire-prone vegetation types, fire also promotes diversity within fire-prone vegetation types. Given that fire-adapted/exapted (see Bradshaw *et al.*, 2011) species vary in their responses to fire and the time frames over which these responses manifest, fire-prone vegetation can display a pronounced postfire succession, usually characterized by a net decline in apparent species richness with time since fire and by temporal turnover in plant species composition and abundance (e.g. Kruger and Bigalke, 1984; Hoffman *et al.*, 1987; Guo, 2001; Slingsby *et al.*, 2017; He *et al.*, 2019). This temporal dispersion of the prevalence of species following fire has the effect of alleviating interspecific competition and facilitating plot-scale coexistence, with positive consequences for alpha diversity (Cowling, 1987; Thuiller *et al.*, 2007). Moreover, because most fire-prone environments show spatial variation in fire history and/or regime (i.e. pyrodiversity; Martin and Sapsis, 1992; Steel *et al.*, 2021) and because plant species differ with respect to the fire regime characteristics that favour their performance and persistence (e.g. some species are favoured by high fire frequency, others not; Magadzire *et al.*, 2019), fire also enhances beta diversity through the provision of diverse, spatially dispersed fire niches (Martin and Sapsis, 1992; He *et al.*, 2019). These patterns are consistent with the generally positive effect of environmental heterogeneity on species richness (Ricklefs, 1977; Kerr and Packer, 1997; Stein *et al.*, 2014; Udy *et al.*, 2021).

Although postfire change in plant community composition and richness is well documented, its proximate drivers remain poorly explored. Consistent with a view that postfire species turnover reflects differential adaptation/exaptation of species to changing conditions along the postfire succession sequence (He *et al.*, 2019), we propose that the magnitude of species turnover following fire is determined by the extent to which fire and subsequent regrowth transform plant resource availability. Fire potentially enhances the availabilities of light, soil moisture (Clemente *et al.*, 2005) and soil nutrients (Kutiel and Naveh, 1987; Caon *et al.*, 2014), with these resources commonly declining in availability with time since fire. Which resources are most influential in modulating the floristic impact of fire, however, i likely to vary from one system to another. In light-limited tall conifer forests, for example, the role of fire in opening canopy gaps and thus enhancing light availability might be of principal importance in driving species turnover (e.g. Spies and Franklin, 1989), whereas in open sclerophyll shrublands on low-nutrient soils, nutrient augmentation associated with ash deposition might be more influential (e.g. Lambers *et al.,* 2022).

The Cape Floristic Region (CFR) of South Africa has some of the poorest soils globally, with mean soil nitrogen and phosphorus concentrations lower than in any other mediterranean climate region (Stock and Verboom, 2012). Unsurprisingly, vegetation distribution and characteristics in the CFR are strongly influenced by bedrock and soil properties (Specht and Moll, 1983; Campbell, 1986; Cowling *et al.*, 1992; Bergh *et al.*, 2014; but for a contrasting view, see Esler *et al.*, 2015). For example, where the nutritionally impoverished sands derived from quartzitic and calcrete rocks predominantly support sclerophyllous fynbos shrubland, the heavier and more fertile soils derived from shales and granites support grassier, semi-deciduous renosterveld and karroid shrubland. Importantly, because many functional traits, including nutritional traits, of plants show phylogenetic signal, with family- and order-level lineages differing in their functional attributes (e.g. Stock and Verboom, 2012; Cornwell *et al.*, 2014; Verboom *et al.*, 2017), these vegetation types are differentiated floristically at the levels of genus, family and order (Bergh *et al.*, 2014). Where fynbos is dominated by lineages having low-nutrient adaptations/exaptations, such as woodiness, sclerophylly, low tissue nutrient concentrations, low growth rate and specialized root systems (e.g. Ericaceae, Proteaceae and Restionaceae; reviewed by Cramer *et al.*, 2014), succulent karoo and renosterveld lineages are characterized by adaptations/exaptations to seasonal aridity, including annualness, succulence, geophytism, leaf deciduousness, high tissue nutrient concentrations and high growth rate (e.g. Aizoaceae, Asteraceae and Poaceae; Verboom *et al.*, 2004, 2017; Cramer *et al.*, 2014). Despite a pronounced difference in the fertility of fynbos vs. renosterveld soils (Specht and Moll, 1983; Campbell, 1986; Cramer *et al.*, 2019*b*), a surprisingly large number of taxa straddle the fynbos and renosterveld floras (Bergh *et al.*, 2014; Verboom *et al.*, 2014), resulting in the latter being termed a ‘transitional’ vegetation type (i.e. between fynbos and succulent karoo; Cowling, 1983). We propose that the floristic overlap between fynbos and renosterveld is explained by fluctuations in the availability of nutrients in fynbos (Brown and Mitchell, 1986; Stock and Lewis, 1986), with fire-mediated ash deposition producing a window of opportunity for high-nutrient-adapted taxa that are otherwise more typical of fertile renosterveld and succulent karoo systems.

Here, we test the hypothesis that fynbos communities display a change in species composition and a net decline in species richness with time since fire and that these effects are a consequence of the high-nutrient pulse that manifests immediately in the wake of fire. We discuss the consequences of this phenomenon for community richness (i.e. alpha diversity) in fynbos. We also test the hypothesis that the floristic affinity of fynbos to renosterveld is attributable to the postfire nutrient pulse observed in fynbos, this providing a transient niche for the entry of high-nutrient-adapted renosterveld lineages. To test these ideas, we compare postfire changes in species richness and composition between mountain fynbos shrubland growing on highly infertile, quartzite-derived sands and renosterveld shrubland growing on more fertile, shale-derived clays. We predict that the postfire decline in species richness is more pronounced in fynbos than in renosterveld, reflecting the disappearance of species belonging to high-nutrient-adapted families. We also predict that the floristic similarity (at the family level) of fynbos to renosterveld declines as a function of increasing vegetation age.

## MATERIALS AND METHODS

### Source data

Plant community composition data were obtained from two relevé-based vegetation surveys, one based in the former Cape of Good Hope Nature Reserve (Taylor 1969; hereafter referred to as ‘Cape Point’) and the second on Signal Hill (Joubert and Moll, 1992; hereafter referred to as ‘Signal Hill’). Both study areas are located on the Cape Peninsula, where they are situated <70 km apart and form part of the Table Mountain National Park. The vegetation of Cape Point comprises largely mesic fynbos (Peninsula Sandstone Fynbos and Hangklip Sand Fynbos; Rebelo *et al.*, 2006) growing on acid sands derived from Table Mountain Group quartzites, whereas the vegetation of Signal Hill comprises principally renosterveld (Peninsula Shale Renosterveld; Rebelo *et al.*, 2006) growing on clay-rich soils derived from Malmesbury Group shales. The coastal margin at Cape Point, however, supports two non-fynbos vegetation types, namely Cape Flats Dune Strandveld (Rebelo *et al.*, 2006) and Cape Seashore Vegetation (Mucina *et al.*, 2006). Although the fertility of soils at Cape Point and Signal Hill has not been quantified, the greater fertility of shale-derived, renosterveld soils relative to quartzite-derived, fynbos soils is well documented (Specht and Moll, 1983; Campbell, 1986; Cramer *et al.*, 2014, 2019*b*). Although we would ideally have included additional fynbos and renosterveld sites in this study, this was not possible owing to the scarcity of surveys giving estimates of vegetation age, and of renosterveld surveys generally.

The studies by Taylor (1969) and Joubert and Moll (1992) used a 10 m × 5 m relevé or plot as a survey unit, with the vegetation in surveyed plots ranging in age from 1 to 40 years in the Cape Point data set and from 1 to 20 years in the Signal Hill data set. In the case of the Cape Point plots, vegetation age had been estimated using counts of annual growth nodes of proteaceous shrubs (Hall, 1959; Taylor 1969), whereas for the Signal Hill plots they had been inferred using City of Cape Town fire records (Joubert 1991: appendix 7). Neither study detailed the characteristics of the most recent fires. To facilitate comparison between the two data sets and because we were interested primarily in successional change over a typical fynbos inter-fire interval (10–20 years; Kraaij *et al.*, 2014), we excluded 12 plots from the Cape Point data set whose postfire vegetation age exceeded 20 years. We also excluded 17 plots located <500 m from the coast, because they do not contain typical fynbos communities, presumably on account of their soils being enriched by marine deposits (Nyaga *et al.*, 2015). Consequently, our Cape Point data set comprised 59 plots containing a total of 336 species, with the number of species per plot ranging from 6 to 79 and having a mean of 40.9 (Supplementary Data Fig. S1A). Although the Cape Point plots were resurveyed in 1996 (Privett *et al.*, 2001), we elected to use the original survey data because only 81 of the original 100 plots were relocated in the 1996 survey and because the original survey captured a broader spread of vegetation ages, including several recently burnt plots. For Signal Hill, we used only plots associated with Subcommunity 1.1, as designated by Joubert and Moll (1992), because the area occupied by Subcommunity 1.2 had been planted with pines until 1976, after which it had been cleared and protected from fire. Consequently, our Signal Hill data set comprised 35 plots containing a total of 174 species, with the number of species per plot ranging from 29 to 73, with a mean of 46.4 (Supplementary Data Fig. S1B). Unfortunately, the vegetation in most plots was rather old (>12 years), with only two plots of age 1 year.

Both source studies recorded species data as ordinal, Braun-Blanquet cover–abundance classes, which are not conducive to sampling standardization using taxon sampling (rarefaction) curves. Therefore, we determined species richness as the number of species recorded in each plot, regardless of their abundance. We acknowledge that, in so doing, what we term species richness is more correctly species density (i.e. number of species per unit area) *sensu*Gotelli and Colwell (2001). We also acknowledge that these are once-off surveys and that we rely on patterns of variation across spatially dispersed plots of unequal age as a substitute for time.

### Postfire changes in species richness and composition

The relationship of species richness to postfire vegetation age in both fynbos (Cape Point) and renosterveld (Signal Hill) was assessed using ordinary least squares regression, as implemented in R v.4.2.1 (R Core Team, 2021), across the set of plots in each study area. To determine which plant families are responsible for any observed changes in overall species richness with time since fire, we assessed the species richness–vegetation age relationship separately for each plant family occurring in ≥20 plots in either vegetation type, with this prevalence threshold being applied to ensure sufficient statistical power to enable detection of a relationship. This was done separately for the Cape Point and Signal Hill plots.

To probe further which lineages dominate early vs. late postfire succession fynbos communities at Cape Point, we determined, for each species, the mean age of the vegetation in all sites in which it was recorded. Using functions in ape v.5.6-2 (Paradis and Schliep, 2019) and phytools v.1.0-3 (Revell, 2012), maximum likelihood ancestral character state estimation was then used to map this variable on a phylogenetic tree generated with V.PhyloMaker2 (Jin and Qian, 2019, 2022). We also used the phylosig function in phytools to assess whether this variable displays significant phylogenetic signal relative to *λ* = 0 (Pagel, 1999).

For each data set, we then used ordinary least squares regression and phylogenetically generalized least squares regression, as implemented in phylolm v.2.6.2 (Ho and Ané, 2014), to assess whether the sign and strength of the species richness–vegetation age relationship shown by different plant families is related to their foliar nutrient concentrations. In these analyses, the response variable was the species richness–vegetation age correlation (*r*) and the predictor the mean foliar concentration of nitrogen [N], phosphorus [P] or potassium [K]. Given that the latter variables were based on foliar nutrient concentration data reported by Verboom *et al.* (2017), these analyses were necessarily confined to the ten high-prevalence families for which Verboom *et al.* (2017) provided such data, i.e. Asteraceae, Cyperaceae, Ericaceae, Fabaceae, Iridaceae, Rosaceae, Proteaceae, Restionaceae, Rutaceae and Scrophulariaceae. Although the data provided by Verboom *et al.* (2017) are not based on samples from Cape Point or Signal Hill, or even from species occurring at these localities, the existence of strong phylogenetic signal in foliar nutrient traits, especially foliar [P] and [K] (Verboom *et al.*, 2017), validates the use of trait means derived from these data as broad general proxies for family-specific foliar nutrient concentrations. Phylogenetic regression was done using a pruned version of the phylogeny presented by Verboom *et al.* (2017).

Finally, to test the prediction that postfire changes in species composition are more pronounced in nutrient-poor fynbos than in more fertile renosterveld, we used a paired-sample *t* test to compare the strength of the species richness–vegetation age correlation across the six families common to the Cape Point and Signal Hill data sets (i.e. Asteraceae, Fabaceae, Iridaceae, Poaceae, Rubiaceae and Scrophulariaceae).

### Postfire vegetation age and the fynbos–renosterveld floristic relationship

To test the hypothesis that the floristic affinities of fynbos to renosterveld reflect the presence of high-nutrient-adapted elements (families) in the early postfire period, we used non-metric multidimensional scaling (NMDS) with Bray–Curtis (Sørenson) dissimilarity, as implemented in vegan v.2.6-2 (Oksanen *et al.*, 2022), to quantify patterns of family-level compositional similarity across the full set of sites at both Cape Point and Signal Hill. NMDS was preferred over other ordination methods (e.g. principal components analysis) because it is comparatively assumption free and has been shown generally to outperform other methods in recovering community similarity patterns (Fasham *et al.*, 1977). In our data set, a high frequency of zeros introduces a zero-bounding issue, resulting in violation of the principal components analysis assumption of linearity. We applied NMDS to a site × family community matrix, in which cell entries describe the number of species representing a particular family at a particular site. We predicted that recently burnt fynbos plots would occupy positions in ordination space closer to the renosterveld plots than older fynbos plots. To assess this statistically, we tested whether the fynbos (i.e. Cape Point) plots displayed a relationship between postfire vegetation age and their score on the first multidimensional scaling axis (MDS1), which describes the separation of fynbos and renosterveld plots.

## RESULTS

### Postfire changes in species richness and composition

**Fig. 1.**
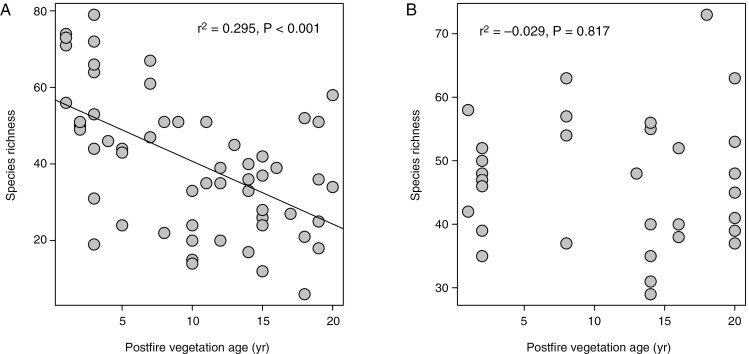
Relationships of species richness to postfire vegetation age across the Cape Point fynbos plots (A) and the Signal Hill renosterveld plots (B).

**Fig. 2.**
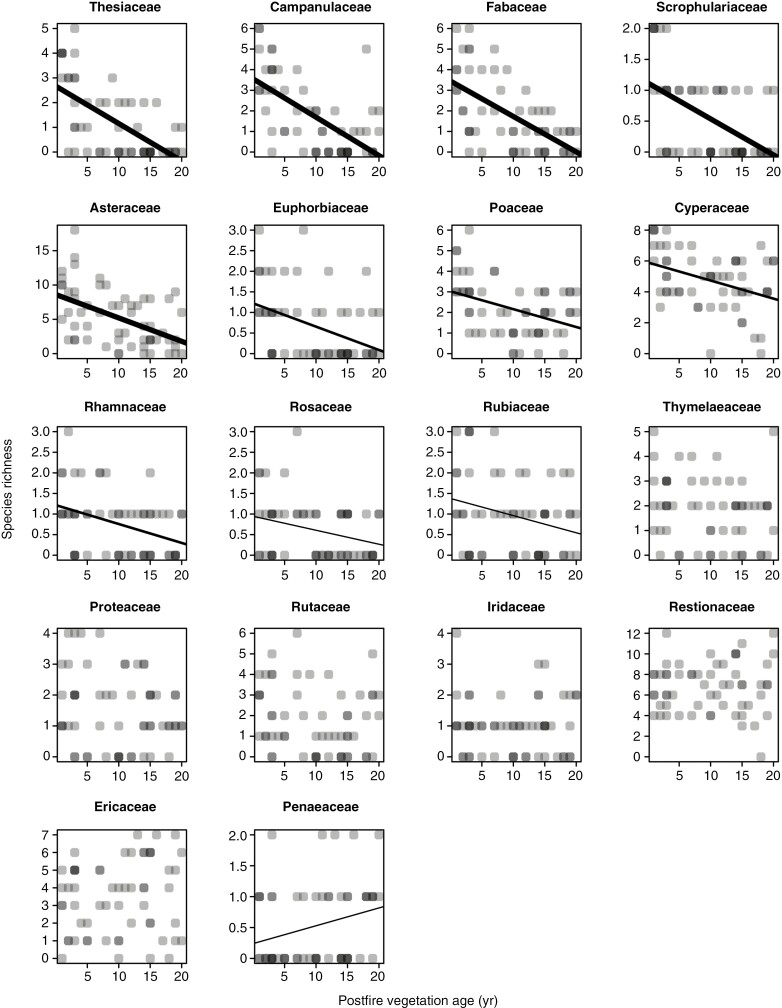
Relationships of species richness to postfire vegetation age across the Cape Point fynbos plots, by family. Only families present in ≥20 plots are included. Given that symbols have a transparent fill, dark points represent instances of multiple overlapping points. The significance of relationship is indicated by line thickness, with thin lines representing *P* < 0.05, medium lines *P* < 0.01 and thick lines *P* < 0.001.

**Fig. 3.**
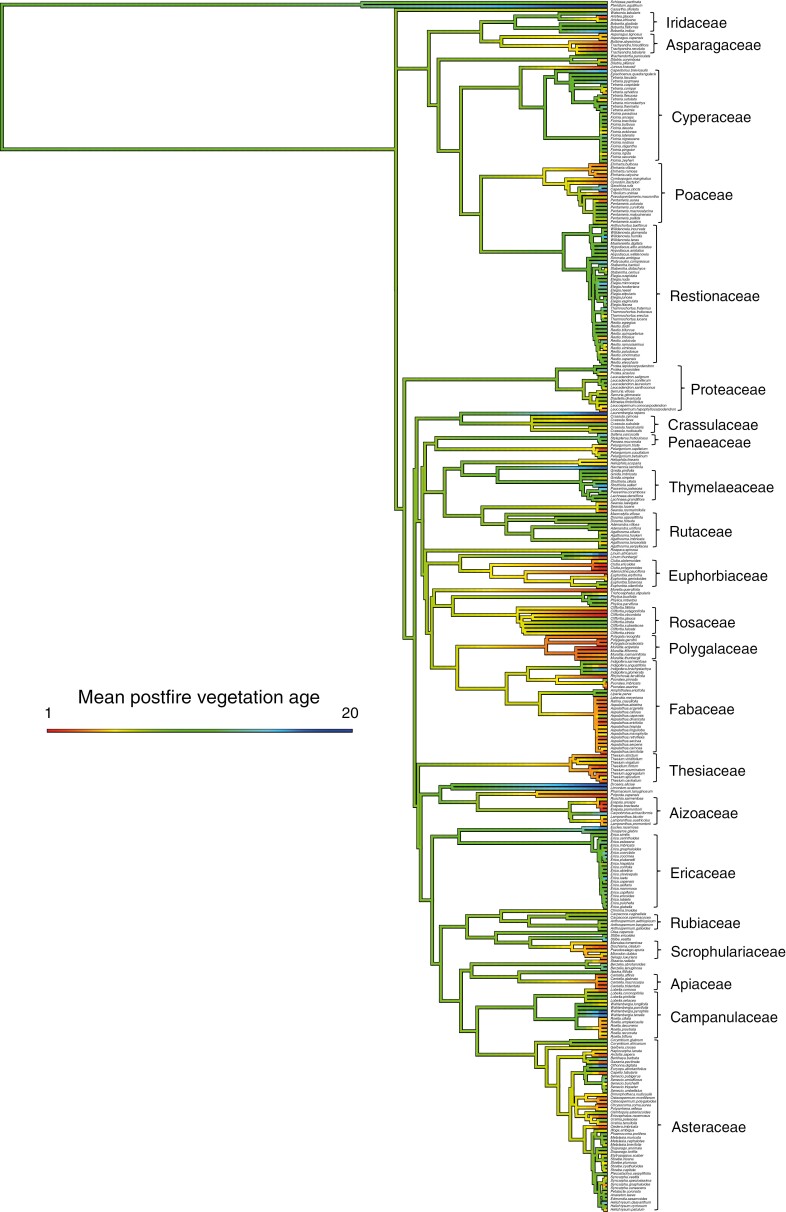
Maximum likelihood phylogenetic reconstruction of the mean age of the vegetation occupied by the Cape Point species. Branch shading indicates vegetation age association, with red and blue, respectively, indicating an association with young (i.e. recently burnt) and old vegetation. Important family-level lineages are indicated on the right of the tree.

The Cape Point fynbos plots display a strong decline in overall species richness with time since fire (Fig. 1A), with this pattern reflecting declines in the species richness of 11 of 18 families meeting our prevalence threshold (Fig. 2), including several that are prominent in renosterveld vegetation, both at Signal Hill and elsewhere. Examples of the latter include Campanulaceae, Fabaceae, Scrophulariaceae, Asteraceae, Euphorbiaceae and Poaceae. In contrast, many of the more typical fynbos-specialist taxa, including Proteaceae, Diosmeae (Rutaceae), Restionaceae, Ericaceae and Penaeaceae, show no evidence of declining species richness, with Penaeaceae even showing an increase in species richness with time since fire. Consistent with these patterns, a reconstruction of the mean age of the vegetation occupied by each of the Cape Point species (Fig. 3) reveals significant phylogenetic signal (*λ* = 0.632, *P* < 0.001), with lineages showing strong postfire species richness declines (Fig. 2) also reflecting an association with younger vegetation. Although they are not represented in Fig. 2 because they fail to meet our prevalence threshold, Aizoaceae, Apiaceae and Crassulaceae also fall into this category (Fig. 3).

**Fig. 4.**
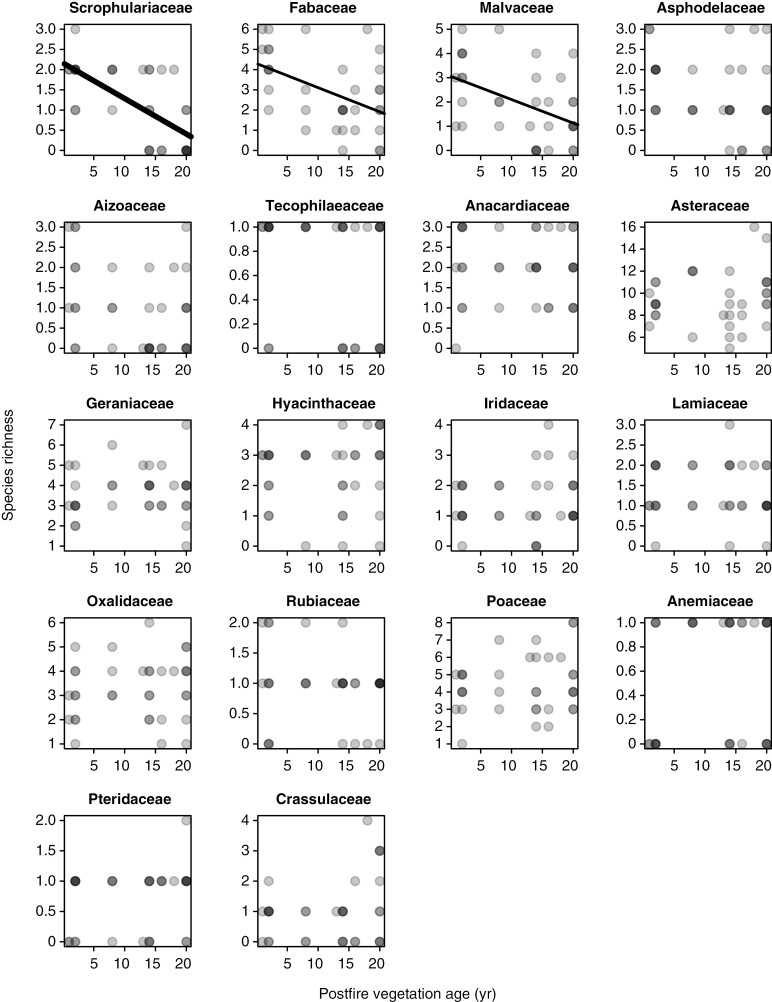
Relationships of species richness to postfire vegetation age across the Signal Hill renosterveld plots, by family. Only families present in ≥20 plots are included. Given that symbols have a transparent fill, dark points represent instances of multiple overlapping points. The significance of relationship is indicated by line thickness, with thin lines representing *P* < 0.05, medium lines *P* < 0.01 and thick lines *P* < 0.001.

**Fig. 5.**
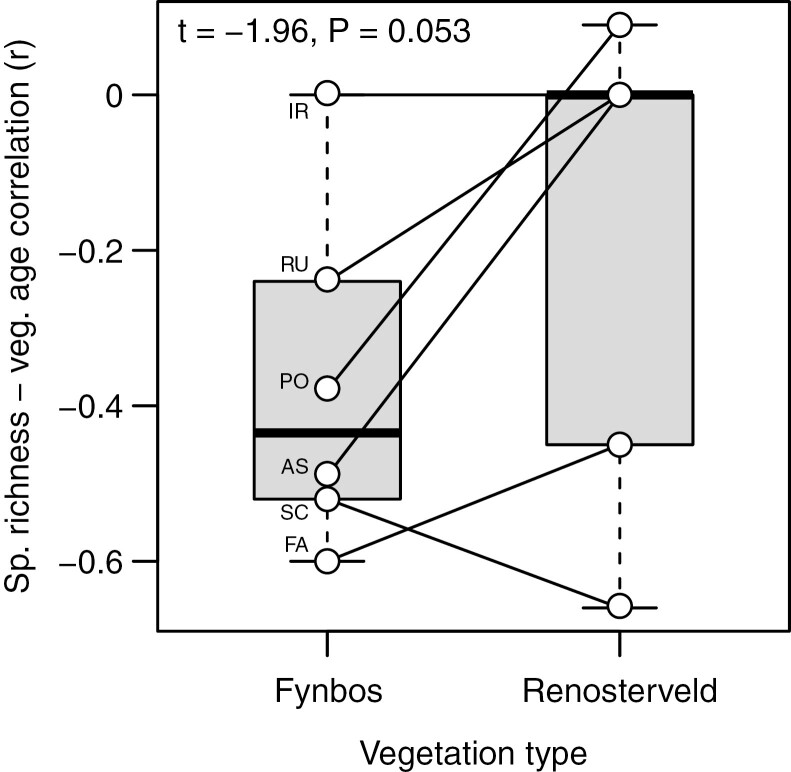
Box-and-whisker comparison, by family, of the species richness–vegetation age correlation, for the Cape Point fynbos (Fig. 2) and Signal Hill renosterveld (Fig. 4) plots. The six families included, by virtue of their presence in ≥20 plots at both Cape Point and Signal Hill, are as follows: AS, Asteraceae; FA, Fabaceae; IR, Iridaceae; PO, Poaceae; RU, Rubiaceae; SC, Scrophulariaceae. The whiskers depict the minimum–maximum ranges, the boxes the 0.25–0.75 interquartile ranges, and the dark bar the median, across the six families.

In contrast to the Cape Point plots, and despite declines being apparent in Scrophulariaceae, Fabaceae and Malvaceae (Fig. 4), the Signal Hill renosterveld plots show no decline in overall species richness with time since fire (Fig. 1B). This suggests that the impacts of fire on species richness and community composition are less pronounced in renosterveld than in fynbos, an inference which is further supported by the species richness–vegetation age correlation being generally weaker in renosterveld than in fynbos, when compared across the six families common to both data sets (Fig. 5).

**Fig. 6.**
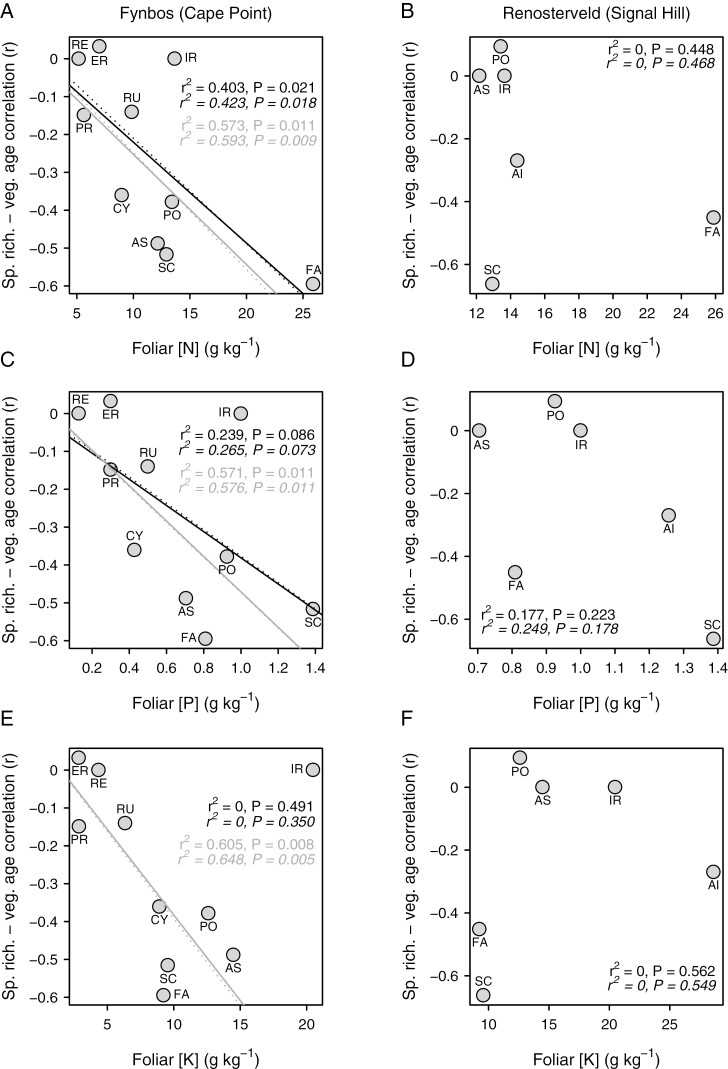
Relationship of the species richness–vegetation age correlation to mean foliar nitrogen (A, B), phosphorus (C, D) and potassium (E, F) concentrations, across families present in ≥20 plots at Cape Point (left panels; *n* = 10) and Signal Hill (right panels; *n* = 6). Family codes are as follows: AS, Asteraceae; CY, Cyperaceae; ER, Ericaceae; FA, Fabaceae; IR, Iridaceae; PO, Poaceae; PR, Proteaceae; RE, Restionaceae; RU, Rutaceae; SC, Scrophulariaceae. Regression lines are fitted using both ordinary least squares (black lines) and phylogenetically generalized least squares (grey lines), with the outlying Iridaceae (IR) both included (solid lines) and excluded (dotted lines).

Besides differing between two vegetation types, the species richness–vegetation age correlation is negatively related to foliar [N], [P] and [K] across families in the Cape Point (fynbos) plots, at least with the exclusion of geophytic Iridaceae, which is a consistent outlier (Fig. 6A, C, E). Although this relationship is robust to the inclusion of Iridaceae in the case of [N] (Fig. 6A), in the case of [P] and [K] it results in a loss of significance, although only marginally so in the case of [P] (Fig. 6C). In the case of [N], the relationship is also robust to the exclusion of Fabaceae (ordinary least squares regression: *r*^2^ = 0.668, *P* = 0.008; phylogenetically generalized least squares regression: *r*^2^ = 0.686, *P* = 0.007). In contrast to the situation at Cape Point, the species richness–vegetation age correlation is unrelated to foliar nutrient concentration across families in the Signal Hill (renosterveld) plots (Fig. 6B, D, F).

### Postfire vegetation age and the fynbos–renosterveld floristic relationship

**Fig. 7.**
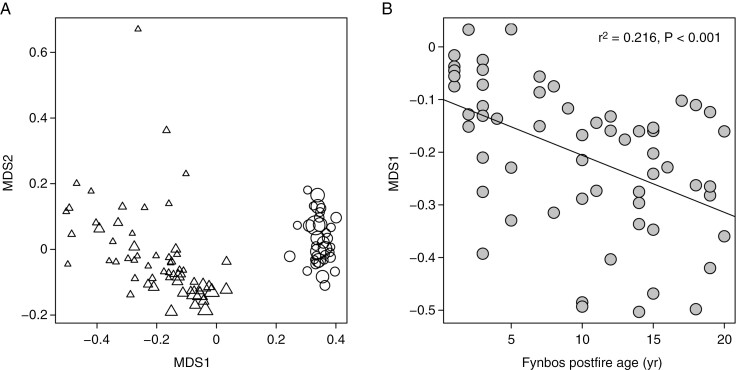
(A) Non-metric multidimensional scaling biplot extracted from a site × family community matrix comprising both the Cape Point fynbos (triangles) and Signal Hill renosterveld (circles) plot data. Cell entries in the input matrix describe the number of species representing a particular family at a particular site, and between-plot distances are based on Bray–Curtis dissimilarity. Symbol size describes postfire vegetation age, with larger symbols representing recently burnt plots. The associated stress value is 0.093. (B) Relationship of the MDS1 score (from panel A), describing the family-level compositional similarity of the Cape Point fynbos plots to the Signal Hill renosterveld plots (high values indicate greater similarity), as a function of postfire fynbos vegetation age.

Application of NMDS to a matrix recording the numbers of species from each family observed in each plot across the combined data sets yields a two-dimensional biplot with a stress value of 0.093, and in which the fynbos and renosterveld plot clusters separate out along MDS1 (Fig. 7A). Consistent with our second hypothesis, younger plots in the fynbos cluster tend to be situated closer to the renosterveld cluster (Fig. 7A; higher MDS1 scores), with a significant negative relationship between MDS1 score and vegetation age, across the fynbos plots, identifying this pattern as significant (Fig. 7B).

## DISCUSSION

Our data demonstrate pronounced changes in fynbos plant community composition with time since fire, with some families showing marked declines in species richness and others not, and with overall species richness experiencing a net decline with time since fire. These patterns are more pronounced in fynbos than in renosterveld. Although we are conscious of our data being drawn from only a pair of sites (Cape Point and Signal Hill), the generality of these patterns is corroborated by our personal field observations. It is also supported by the literature, which reveals postfire species richness declines to be a feature of many fire-prone systems (e.g. Kruger and Bigalke, 1984; Hoffman *et al.*, 1987; Guo 2001; He *et al.*, 2019) and postfire changes in family-level composition to be a common feature of phosphorus-constrained, fire-prone vegetation (e.g. Lambers *et al.*, 2022). Echoing patterns reported here, Kruger (1987: pp. 72–74) identified geophytes and postfire ephemerals as the most important contributors to elevated postfire species richness in fynbos, with these elements being drawn principally from Aizoaceae, Asteraceae, Campanulaceae, Crassulaceae, Fabaceae, Iridaceae, Scrophulariaceae and Thesiaceae.

We hypothesized that postfire changes in fynbos plant community composition and richness are powered principally by changes in soil nutrient availability with time following fire (Brown and Mitchell, 1986; Stock and Lewis, 1986), and three patterns support this idea. First, the postfire decline in overall species richness is apparent only in the fynbos vegetation at Cape Point, not in the renosterveld vegetation at Signal Hill. Although soil fertility at these sites has not been measured, the greater fertility of shale-derived, renosterveld soils relative to quartzite-derived, fynbos soils is well documented (Specht and Moll, 1983; Campbell, 1986; Cramer *et al.*, 2014, 2019*b*), and we propose that the stronger postfire species richness spike observed in fynbos is attributable to the greater nutritional impact of ash deposition there. Second, the decline in overall species richness observed in fynbos is replicated across multiple plant families, with the species richness–vegetation age correlation being generally negative and stronger in fynbos than in renosterveld. Third, at Cape Point the species richness–vegetation age correlation is related to family-level variation in foliar nutrient concentrations (ionome *sensu*Salt *et al.*, 2008), a trait showing strong phylogenetic signal and apparently having a strong genetic basis (Broadley *et al.*, 2004; Kerkhoff *et al.*, 2006; Verboom *et al.*, 2017). Given the importance of foliar nutrient concentrations as determinants of plant growth potential in conditions of high nutrient availability (Elser *et al.*, 2000; Wright *et al.*, 2004), this pattern almost certainly reflects the ability of some lineages, notably Thesiaceae, Campanulaceae, Fabaceae, Scrophulariaceae, Asteraceae, Euphorbiaceae, Poaceae, Cyperaceae, Aizoaceae and Crassulaceae, to respond to the postfire nutrient pulse observed in fynbos, and their inability to cope with the low-nutrient conditions that characterize later postfire succession. Although Verboom *et al.* (2017) identified some of these families as nutritional generalists, in contrast to the low-nutrient specialists that dominate mature fynbos, they might, alternatively, be high-nutrient specialists. This is, perhaps, more consistent with the lack of a clear relationship between specialization and disturbance response in plants (Vázquez and Simberloff, 2002).

Although we argue for a principal role of nutrient augmentation in stimulating postfire changes in species richness and composition in fynbos, we do not exclude a role for other resources, such as light, whose availability is also influenced by fire. For example, mechanical defoliation, which increases light but not nutrient availability, has been shown to enhance the richness, density and flowering of geophytes in fynbos, albeit not to the same extent as fire (Verboom *et al.*, 2002; Vlok 2020). Interestingly, in geophytic *Ehrharta* (Poaceae) postfire flowering levels are achieved only with a combination of fertilization and defoliation (Verboom *et al.*, 2002), which suggests that the responsiveness of high-nutrient-adapted plants to nutrient augmentation is contingent on a simultaneous increase in light availability.

Beyond having higher foliar nutrient concentrations, the families that dominate early postfire succession fynbos possess other traits that reflect a high demand of soil nutrients, especially P and K. For example, the ability of Fabaceae to acquire N symbiotically offers significant benefits, but only in conditions of high P availability (Power *et al.*, 2010, 2011). In the presence of low P, a trade-off between investment in N_2_ fixation and in traits enabling the acquisition of sparse P might compromise the ability of legumes to acquire P (Power *et al.*, 2010; Cramer *et al.*, 2014), thereby upsetting the N:P stoichiometry of these plants (Güsewell, 2004; Neugebauer *et al.*, 2018). This might explain the general failure of Fabaceae species to persist beyond the earliest stages of postfire succession, as P becomes scarcer. Succulence, as exemplified by Aizoaceae, Crassulaceae and Euphorbiaceae, also appears to carry a high soil nutrient demand. In part, this might be attributable to the low and sporadic availability of soil moisture in environments favoured by succulents, this limiting opportunities for nutrient uptake and necessitating an association with more fertile soils (Ripley *et al.*, 2013). In addition, the tendency of Aizoaceae and Crassulaceae to maintain high foliar concentrations of calcium, magnesium, potassium and sodium (White *et al.*, 2016; Verboom *et al.*, 2017), possibly as an anti-herbivore defence (Franceschi and Nakata 2005), might necessitate an association with conditions of high soil fertility. There is also evidence to show that high tissue concentrations of inorganic salts are a requirement for CO_2_ fixation in succulents using the crassulacean acid metabolic pathway (Bloom, 1979), although this might better explain the high postfire incidence of Crassulaceae than that of Aizoaceae (see Ripley *et al.*, 2013). Although the strong association of hemiparasitic Thesiaceae with the postfire nutrient flush seems surprising, given the apparent ability of these plants to source both carbon and minerals from their hosts (Fer *et al.*, 1993, 1994; Luo and Guo, 2010; Giesemann and Gebauer, 2022), the low nutrient concentrations in the tissues of potential host species might limit the efficacy of parasitism as a nutrient acquisition strategy in fynbos, especially later in the postfire succession sequence. Finally, although the nutrient dependence of Asteraceae, Campanulaceae, Cyperaceae, Poaceae and Scrophulariaceae has been little studied, a high incidence of herbaceousness and annuality in these families, paired with the production of dense inflorescences in Asteraceae, Cyperaceae, Poaceae and some Scrophulariaceae, suggests a general proclivity for fast growth and increased dependence on seed-mediated persistence (e.g. Garnier, 1992; Garnier and Vancaeyzeele, 1994; Verboom *et al.*, 2004, 2012), with a need to bear the high nutrient costs that these incur (Bloom *et al.*, 1985). Indeed, the nutrient cost associated with seed production is predicted generally to be high in short-lived plants whose persistence across fire cycles depends on the establishment of soil-based seed banks (e.g. many Fabaceae, Thesiaceae).

In contrast to the lineages discussed above, several of the families that dominate the later stages of postfire succession possess traits that facilitate the acquisition of sparse nutrients from soils that are critically infertile because nutrients, besides being scarce, are also bound and thus inaccessible (Lambers *et al.*, 2011; Cramer *et al.*, 2014; Raven *et al.*, 2018). These traits include the formation in Proteaceae, Restionaceae and schoenoid Cyperaceae of short-lived cluster roots, which can mobilize and rapidly absorb insoluble inorganic P (Lambers *et al.*, 2006), and the formation in Ericaceae of associations with ericoid ectomycorrhizas, which are capable of hydrolysing organic forms of P (Smith and Read, 2008; Raven *et al.*, 2018). Significantly, although Cyperaceae display a decline in species richness with time since fire, this pattern reflects a decline in the species richness of non-schoenoid sedges, which lack root specializations. The schoenoid sedges, which commonly possess dauciform roots (Shane *et al.*, 2006), show no such decline (Supplementary Data Fig. S2).

Beyond adaptations for enhanced nutrient acquisition, most of the lineages that dominate mature fynbos also possess traits that enhance nutrient retention. These include the production of small leaves with low specific leaf area (Ordoñez *et al.*, 2009; Yates *et al.*, 2010), as observed in fynbos Rhamnaceae (i.e. *Phylica*), Rutaceae (i.e. *Diosmeae*) and Penaeaceae (Onstein *et al.*, 2014; Onstein and Linder, 2016), or leaf loss and the transfer of photosynthetic function to the high-shoot-mass-per-area culms, as observed in Restionaceae (Ehmig *et al.*, 2019). In all these plants, the functional significance of producing low-specific leaf area/high-shoot-mass-per-area photosynthetic tissues relates to the greater longevity of these tissues (Wright *et al.*, 2004), this facilitating nutrient retention and functioning as an adaptation to low soil fertility (Cramer *et al.*, 2014). Underground storage structures, as observed in Iridaceae, represent an alternative mechanism for the resorption and retention of nutrients assimilated during the postfire nutrient flush and initially stored in the leaves. In Iridaceae and other fynbos geophytes, annual corm replacement is prioritized over other activities, including flowering, with the developing daughter corm being the dominant nutrient sink (Verboom *et al.*, 2002; Vlok, 2021) and with resorption of nutrients from the senescing leaves into the corm (or bulb) being exceptionally efficient (Ruiters *et al.*, 1993, 1994). Thus, although these species continue to produce a few leaves every year through the postfire succession sequence to replenish carbohydrate stocks, a high resorption efficiency ensures that nutrient loss is minimal and explains the ability of Iridaceae to produce unusually high-nutrient leaves in nutritionally impoverished, mature fynbos environments (Fig. 5). Significantly, sexual reproduction (flowering and fruiting) in fynbos geophytes typically takes place only in the first year or two after fire (Levyns, 1929; Linder, 1990; Le Maitre and Brown, 1992; Vlok, 2020), apparently as a plastic response to increased light and nutrient availability (Verboom *et al.*, 2002; Vlok, 2020).

Given the greater fertility of renosterveld relative to fynbos, the observation that renosterveld-associated plant families dominate the early postfire window in fynbos and disappear as the vegetation matures provides further support for the idea that these changes are nutritionally driven. It also supports the hypothesis that the floristic similarities between fynbos and renosterveld owe their existence to the role of fire in providing a transient high-nutrient niche in fynbos. Given that renosterveld and fynbos represent floristically distinct, biome-like entities (e.g. Bergh *et al.*, 2014), the dispersion of floristic elements representing each along a postfire succession sequence identifies fynbos as a composite biome, i.e. a biome comprising two biome-like elements that coexist spatially, but which are temporally dispersed. This perspective departs from the conventional view of biomes as purely spatial entities that are stable at least on shorter, ecological time scales, and which can be mapped (Higgins *et al.*, 2016; Mucina, 2019; Conradi *et al.*, 2020).

Whether or not categorical assignment of species to a biome is useful in this context, the recognition that fire enables the spatial coexistence of floristically distinct assemblages contributes to our understanding of fynbos species richness. Just as spatial heterogeneity promotes landscape-scale coexistence and species richness through the provision of diverse niches (Stein *et al.*, 2014; Udy *et al.*, 2021), so too the effect of fire in generating diverse nutritional niches must enhance species richness, although in this case the temporal rather than spatial dispersion of these niches allows for species richness to be enhanced even at the community scale (alpha diversity).

In this context, it is interesting that the alpha diversity of fynbos is commonly considered to be unexceptional (e.g. Cowling, 1983; Cowling *et al.*, 1992, 1996; Simmons and Cowling, 1996), although Bond (1983), comparing community richness across a range of mediterranean-climate and temperate ecosystems, considered fynbos communities to ‘rank as rich in the world’s vegetation’. Bond also recognized the sensitivity of alpha diversity estimation to the time of sampling, including the inclusion or exclusion of ephemeral life forms, such as annuals and geophytes. Given that estimates of fynbos alpha diversity are typically based on vegetation surveys conducted at a particular point along the postfire succession sequence, and often several years following fire when community richness is in decline, they are likely to underestimate the true alpha diversity. Expanding the analogy of Pausas and Keeley (2014*b*), who compare the multi-year fire cycle in a shrubland ecosystem like fynbos with the annual moisture cycle in a seasonally arid ecosystem, sampling mature fynbos to determine species richness is akin to sampling a seasonally arid ecosystem towards the end of the dry season. This highlights the challenges in detecting and attributing the impacts of global change drivers on species richness and composition in this ecosystem (Slingsby *et al.*, 2017).

Our data suggest that the postfire recovery cycle has a greater impact on apparent species composition and richness in fynbos than in renosterveld. We attribute this to the low nutritional status of fynbos relative to renosterveld soils, which ensures that the nutritional impact of ash deposition is greater in fynbos. Viewed through the lens of classical coexistence theory (MacArthur and Levins, 1967), we suggest that the exceptionally low baseline nutrient content of fynbos soils has the effect of lengthening the nutritional resource axis along which species can differentiate and coexist, thus providing space for low-nutrient extremophiles (mid to late postfire succession) in addition to species that favour more fertile conditions (early postfire succession). This effect might also explain variation in alpha richness between the five mediterranean-climate regions of the world, with the intercepts of regional log(species richness)–log(area) curves (data from Cowling *et al.*, 2015: fig. 2) being positively related (Supplementary Data Fig. S3; *r*^2^ = 0.793, *P* = 0.027) to soil [P] (data from Stock and Verboom, 2012: table 1). In the context of mediterranean-climate vegetation, of course, the association of high species richness with infertile soils has long been appreciated (e.g. Cowling *et al.*, 1996), with different mechanisms being invoked to account for it. Some authors have invoked the emergence of diverse adaptations to nutrient scarcity, particularly phosphate scarcity, in facilitating local species coexistence and thus contributing to species richness (e.g. Lambers *et al.*, 2011, 2018; Laliberté *et al.*, 2014; Zemunik *et al.*, 2015; Hayes *et al.*, 2021), whereas others have invoked the effect of edaphic extremity in accentuating the spatial variability of soil fertility, in addition to the role of fire in generating temporal variability (Cramer and Verboom, in press).

Although our work addresses the impact of fire-modulated nutrient dynamics on fynbos species richness, we note that the mechanism we invoke is applicable to any system in which episodic disturbance affects the availability of a key resource. In tropical rainforest systems, for example, extreme light limitation imposed by a continuous, tiered canopy is interrupted episodically by treefall events, which enable the establishment of light-loving species (Denslow, 1987) and enhance species richness (Strong, 1977; Phillips *et al.*, 1994). Thus, the phenomenon is likely to be general, although the key resources that stimulate species turnover following disturbance might vary between systems.

## Supplementary data

Supplementary data are available at *Annals of Botany* online and consist of the following.

Figure S1: histograms describing the age distribution of the Cape Point fynbos plots (A) and Signal Hill renosterveld plots (B) included in this study. Figure S2: relationships of species richness to postfire vegetation age across the Cape Point fynbos plots, for the non-schoenoid (A) and schoenoid (B) Cyperaceae. Figure S3: relationship of mean community richness (i.e. alpha diversity) to mean soil phosphorus concentration ([P]) across the five mediterranean-type ecosystems of the world.

mcad199_suppl_Supplementary_Figures_S1-S3
