# Supplementary material for: Fire-modulated fluctuations in nutrient availability stimulate biome-scale floristic turnover in time, and elevated species richness, in low-nutrient fynbos heathland
Source: Ann Bot. 2023 Dec 27;133(5-6):819–32. doi: 10.1093/aob/mcad199 (PMC11082518; doi:10.1093/aob/mcad199)
Supplement: mcad199_suppl_Supplementary_Figures_S1-S3 [file mcad199_suppl_supplementary_figures_s1-s3.pdf]

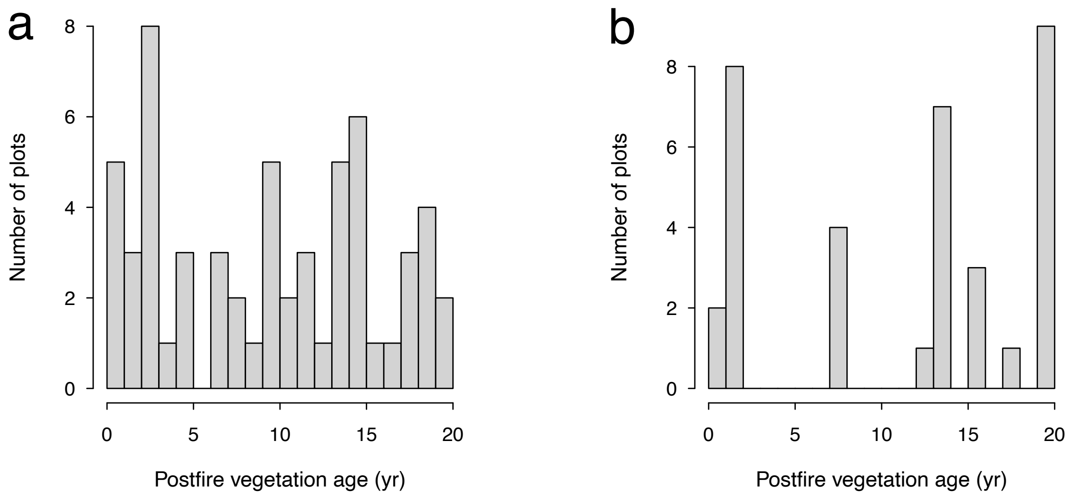

Figure S1. Histograms describing the age distribution of the (a) Cape Point fynbos plots and (b) Signal Hill renosterveld plots included in this study.

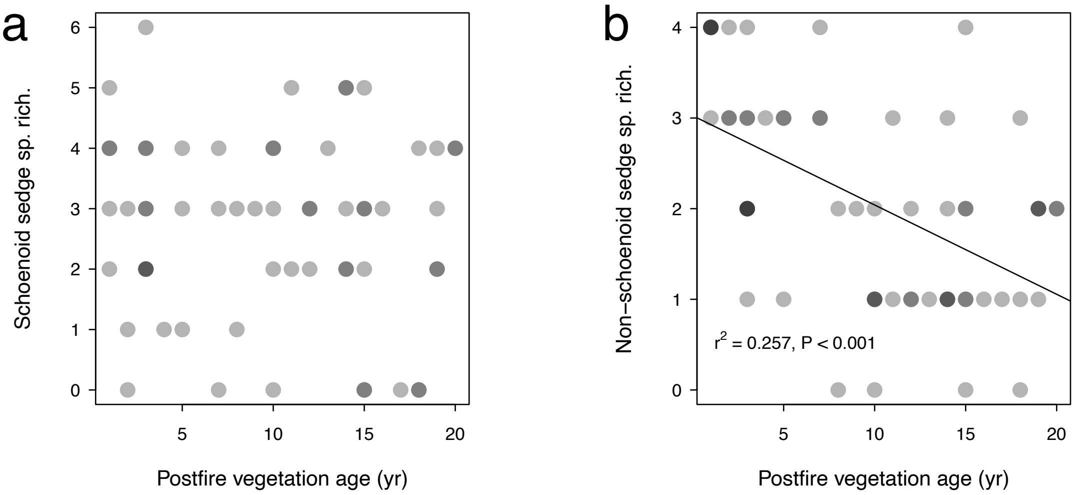

Figure S2. Relationships of species richness to postfire vegetation age across the Cape Point fynbos plots, for the (a) non-schoenoid and (b) schoenoid Cyperaceae. Since symbols have a transparent fill, dark points represent instances of multiple overlapping points. The fitted line has $r^2 = 0.257$, $P < 0.001$.

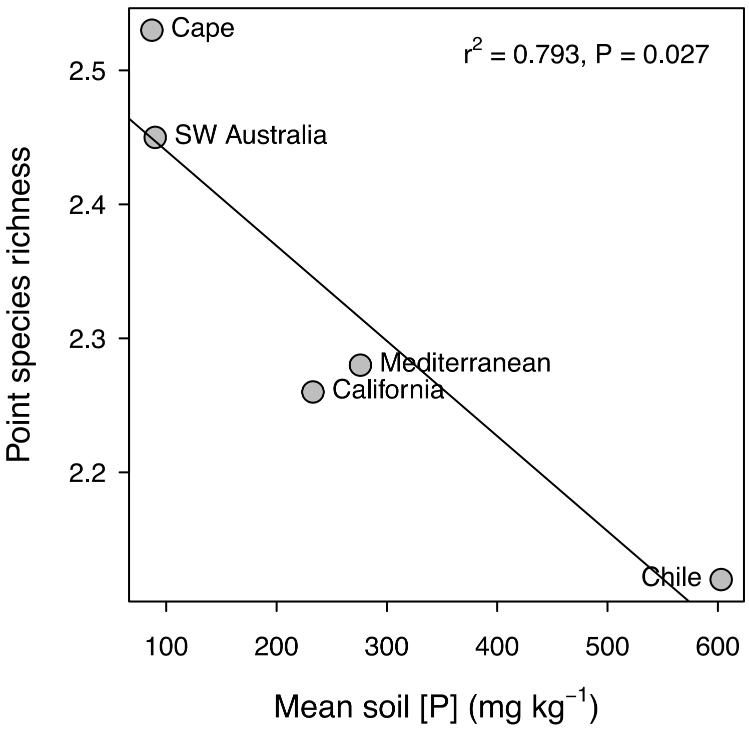

Figure S3. Relationship of mean community richness (i.e., alpha diversity) to mean soil phosphorus concentration [P] across the world's five Mediterranean-type ecosystems. Where the mean community richness of a region is determined as the intercept of the regional log (species richness) - log (area) curve (Cowling et al. 2015: Fig. 2), the mean soil phosphorus concentration [P] is based on data presented by Stock and Verboom (2012).
